# Vertebrojugular Arteriovenous Fistula Following Failed Central Venous Catheter Insertion: A Rare Case

**DOI:** 10.7759/cureus.102054

**Published:** 2026-01-22

**Authors:** Sanhita V Patil, Suresh Phatak, Kajal Mitra, Prashant Onkar, Mayank Rangari

**Affiliations:** 1 Radiodiagnosis, N. K. P. Salve Institute of Medical Sciences and Research Centre and Lata Mangeshkar Hospital, Nagpur, IND

**Keywords:** dsa, iatrogenic vascular injury, mri, vascular malformation, vertebrojugular arteriovenous fistula

## Abstract

Vertebrojugular arteriovenous fistulas (AVFs) are rare vascular anomalies characterized by an abnormal communication between the extracranial vertebral artery and the internal jugular vein. They may arise from trauma, iatrogenic injury, or, rarely, occur spontaneously. We present a 50-year-old male who sustained an iatrogenic injury and developed progressive pulsatile swelling on the right side of the neck, which raised suspicion of an underlying vascular lesion. Subsequent carotid Doppler, MRI with non-contrast angiography, and digital subtraction angiography revealed a vertebrojugular AVF. This case emphasizes the importance of maintaining a high index of suspicion for AVFs in failed central catheter insertions and highlights the effectiveness of early diagnosis in improving patient outcomes.

## Introduction

Vertebral AVFs constitute only about 3% of all acquired traumatic arteriovenous fistulas. In pooled vertebral artery AVF series (which include vertebrojugular variants), traumatic mechanisms account for approximately ~19 % of vertebral AVFs, while ~14 % are attributable to iatrogenic injury from procedures such as central venous catheterization or cervical surgery [1-3]. Vertebrojugular AVFs are rare due to the protected anatomical course of the vertebral artery, with an estimated incidence of <0.1% following neck interventions [3]. Infrequently, they may arise spontaneously in individuals with vessel wall fragility or connective tissue disorders [4], or as part of congenital vascular malformations, though these occurrences are rare [5]. Clinical presentations may include radiculopathy (20-40%), pulsatile tinnitus or cervical bruit (30-50%), and features of vertebrobasilar insufficiency, while high-flow fistulas may lead to cardiac volume overload or venous hypertension if left untreated [3-5].

Historically, open surgical repair was associated with higher morbidity due to difficult surgical exposure and risk of neurovascular injury. With advances in neuroendovascular techniques, endovascular embolization or stent-assisted reconstruction now achieves technical success rates exceeding 85-95%, with lower complication rates and faster recovery, making it the preferred first-line treatment in most cases [5].

We present this case of a vertebrojugular AVF following failed central venous catheterization in an elderly patient to highlight a rare but serious iatrogenic complication and emphasize the importance of early radiologic diagnosis in preventing potentially life-threatening sequelae.

## Case presentation

A 50-year-old male was hospitalized for medical management of an acute illness and was hemodynamically stable at presentation. In anticipation of the need for secure venous access for ongoing intravenous therapy, a right internal jugular vein catheterization was attempted but was unsuccessful and subsequently abandoned, with no immediate complications noted.

Shortly thereafter, the patient developed a gradually progressive, painless, pulsatile swelling on the right side of the neck. There was no history of penetrating or blunt cervical trauma, prior neck surgery, or known vascular malformations. On examination, a soft pulsatile cervical mass with a subtle palpable thrill was noted, raising suspicion of an iatrogenic vascular injury and prompting further imaging evaluation.

**Figure 1:**
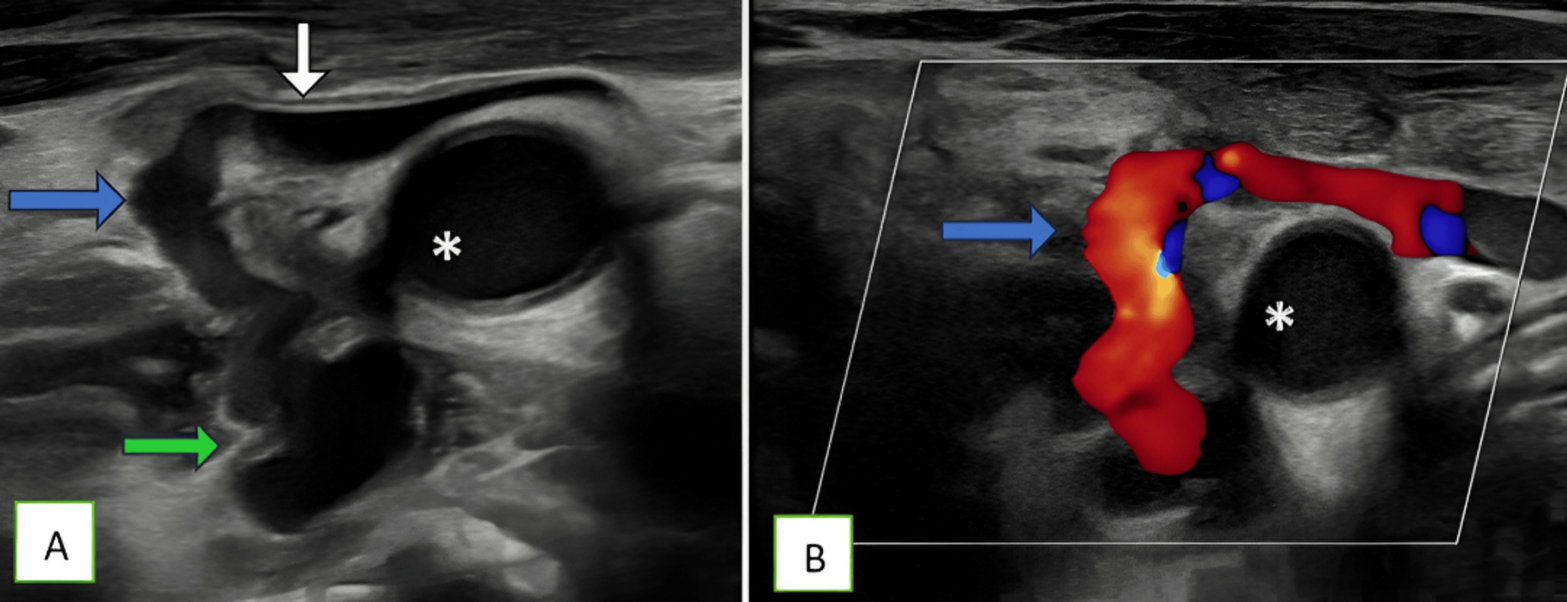
Carotid Doppler ultrasound: (A) grayscale image demonstrating an abnormal fistulous communication (blue arrow) between the right vertebral artery (green arrow) and the right internal jugular vein (white arrow). (B) Color Doppler image showing aliasing artifact at the site of the fistula (blue arrow) * indicates the right common carotid artery

**Figure 2:**
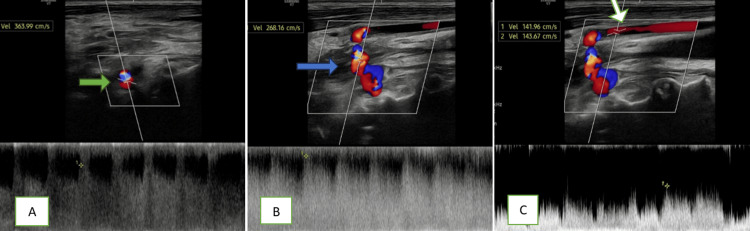
Doppler ultrasound findings: (A) PW spectral analysis demonstrating increased systolic and diastolic velocities in the vertebral artery proximal to the fistula (green arrow). (B) Turbulent, high-velocity flow at the fistula site with aliasing artifact on color Doppler (blue arrow). (C) Arterialized, pulsatile flow within the right internal jugular vein (white arrow) PW: pulsed-wave

A quick bedside carotid Doppler was performed, demonstrating an abnormal fistulous tract between the right internal jugular vein and the vertebral artery, with color Doppler showing aliasing artifact (Figure 1A-1B). Pulsed-wave (PW) spectral study revealed increased systolic and diastolic velocities in the vertebral artery proximal to the fistula; turbulent, high-velocity flow at the fistula site with aliasing artifact; and arterialized and pulsatile flow in the right jugular vein (Figure 2A-2C).

**Figure 3:**
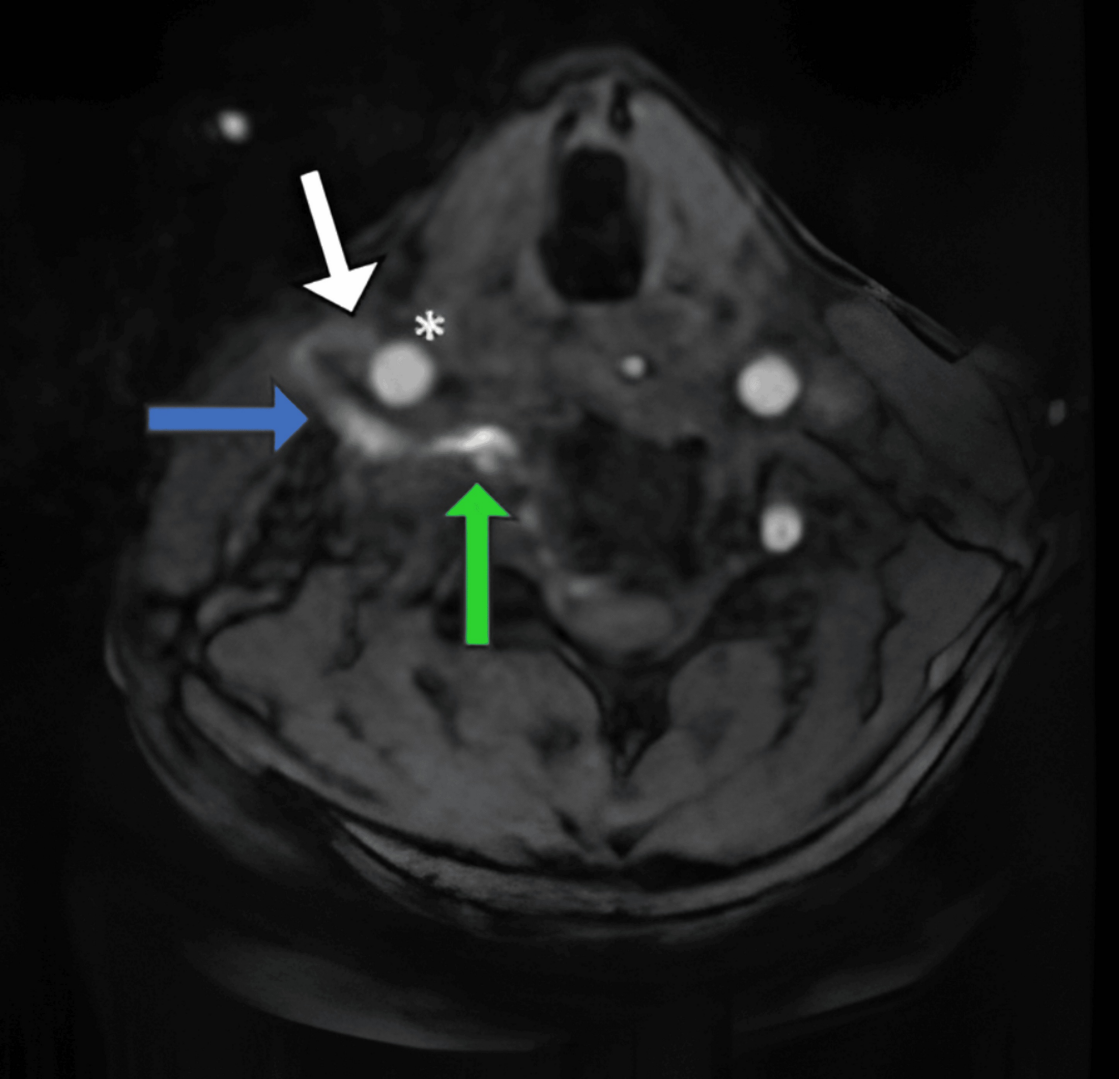
Brain MRI with non-contrast MRA demonstrating a fistulous communication (blue arrow) between the right vertebral artery (green arrow) and the right internal jugular vein (white arrow) * indicates the right common carotid artery MRI: magnetic resonance imaging, MRA: magnetic resonance angiography

Followed by which patient underwent MRI of the brain with non-contrast angiography, which showed a fistulous communication between the right internal jugular vein and the V2 segment of the right vertebral artery at the C3-4 intervertebral level (Figure 3). Loss of flow-related opacification was noted in the V2 segment of the right vertebral artery distal to the level of the fistula.

**Figure 4:**
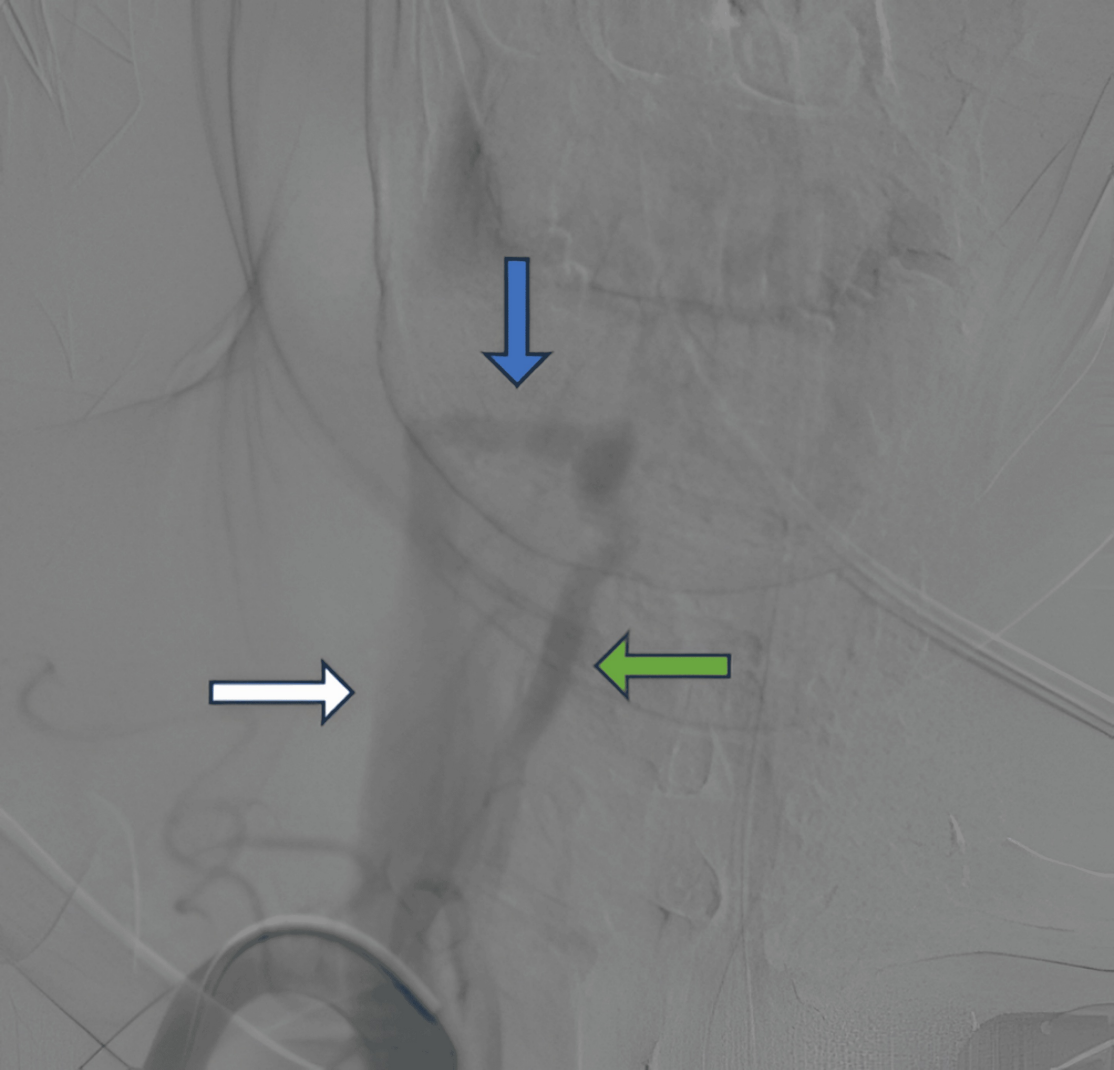
DSA confirming a high-flow right vertebrojugular AVF (blue arrow). The right internal jugular vein (white arrow) and right vertebral artery (green arrow) are shown AVF: arteriovenous fistula, DSA: digital subtraction angiography

Subsequent digital subtraction angiography (DSA) demonstrated early opacification of the right internal jugular vein during the arterial phase, consistent with a high-flow arteriovenous shunt arising from the right vertebral artery, confirming the diagnosis of a right vertebrojugular AVF (Figure 4). The distal V2 segment of the right vertebral artery, beyond the site of the fistula, revealed absent opacification, reflecting diversion of flow into the low-resistance arteriovenous shunt.

Given the high-flow nature of the fistula and the risk of progressive symptoms and potential complications, endovascular occlusion was recommended as the definitive treatment. However, further intervention was deferred as the patient declined consent for the procedure, and the patient was managed conservatively with close clinical follow-up.

## Discussion

Vertebrojugular AVFs are extremely rare due to the deeply protected course of the vertebral artery within the transverse foramina [1,2]. Vertebrojugular and vertebral AVFs are most commonly traumatic in origin, accounting for approximately 70-80% of cases, typically following blunt or penetrating cervical injury or cervical spine fractures [1,3]. Iatrogenic causes contribute around 10-20%, often related to catheterization or cervical procedures [2]. Spontaneous or connective-tissue-related AVFs are uncommon (<5-10%), usually associated with vessel wall fragility or aneurysm rupture [5]. Congenital or malformation-related cases are exceedingly rare (<5%) and generally occur as part of complex vascular malformation patterns.

A multimodality imaging approach, including Doppler ultrasound, MRA, CT angiography (CTA), and confirmatory DSA, is key for correct diagnosis and comprehensive anatomical assessment of vertebrojugular and vertebral AVFs, ensuring precise treatment planning.

Carotid Doppler shows high-velocity turbulent flow, arterialized venous waveform in the internal jugular vein, and loss of normal phasicity, indicating a high-flow arteriovenous shunt [1,2]. On the PW Doppler study, the vertebral artery proximal to the fistula demonstrates a high-velocity, low-resistance waveform. In contrast, the fistula site reveals turbulent flow with an arterialized venous waveform in the internal jugular vein. Distal to the fistula, flow is often dampened with low resistance [6].

CTA typically shows a direct arteriovenous connection with early venous opacification, along with dilated draining veins and possible associated arterial injury such as vessel irregularity or pseudoaneurysm, findings commonly reported in traumatic and iatrogenic vertebral AVFs [1,3,5].

MR angiography (MRA) reveals direct fistulous communication between the vertebral artery and jugular vein with early venous filling, asymmetric venous engorgement, and dilatation of draining veins. MRA findings in vertebral AVFs reported in traumatic cases show similar early venous opacification and abnormal flow-related enhancement [1,4,5].

DSA provides definitive visualization of the AVF, demonstrating single or multiple arterial feeders, rapid shunting, and immediate opacification of the jugular venous system during the arterial phase. DSA also helps identify associated vascular injuries such as pseudoaneurysm or vessel irregularity, which are documented in vertebral artery trauma and AVFs [1,3,5].

The treatment of vertebrojugular and vertebral AVFs is primarily endovascular, with coil embolization, balloon occlusion, or stent-assisted closure as preferred modalities due to their high success rates and low complication rates [1,5]. Surgical intervention is now rarely required and is generally reserved for complex or inaccessible lesions.

The prognosis is excellent when treated early, with most patients showing rapid improvement in symptoms such as pulsatile tinnitus, bruit, or radiculopathy following endovascular closure [1,5]. Delayed diagnosis, however, may result in persistent venous hypertension or neurologic deficits [1,4]. Overall, early recognition and minimally invasive treatment lead to durable fistula occlusion and favorable long-term outcomes, with recurrence being uncommon in treated traumatic and iatrogenic AVFs [3,5].

A recent case report by Mi et al. described an iatrogenic vertebrojugular AVF with an associated vertebral artery pseudoaneurysm following multiple vascular procedures, successfully managed with endovascular stent-grafting [7]. Melge et al. reported a traumatic vertebrojugular AVF following high-velocity blunt cervical trauma, which was treated with a covered stent, achieving fistula occlusion with preservation of vertebral artery flow [8].

Yoshihara et al. described a case of a cervical fracture complicated by a high-flow vertebro-vertebral arteriovenous fistula, which was managed with transarterial coil embolization, resulting in complete closure [9].

Given the possibility of subtle or misleading presentations, several conditions should be considered in the differential diagnosis when evaluating a vertebrojugular AVF, including (1) vertebral-vertebral AVFs, which are typically traumatic or iatrogenic and demonstrate early venous filling with a dilated vertebral venous plexus on CTA, MRA, or DSA; (2) carotid-jugular AVFs, usually post-traumatic or catheter-related, characterized by direct carotid-internal jugular communication, turbulent high-velocity flow, and early venous opacification; (3) vertebral or carotid artery pseudoaneurysms, which appear as focal contrast-filled outpouchings with swirling flow on Doppler (“yin-yang sign”) and can mimic AVFs; (4) craniovertebral or spinal dural AVFs, in which DSA reveals perimedullary flow voids, venous congestion, and a slowly draining dural feeder; and (5) high-flow vascular neck tumors such as paragangliomas or glomus tumors, which exhibit a hypervascular “salt-and-pepper” appearance on MRI and show tumor blush on angiography without a direct arteriovenous shunt.

## Conclusions

Vertebrojugular AVFs are extremely rare, and prompt early diagnosis is crucial, as these lesions can rapidly lead to venous hypertension, neurological deficits, or high-flow complications if not treated. A multimodality imaging approach, using Doppler ultrasound, CTA, MRA, and DSA, the gold standard, ensures accurate detection of the fistula and arterial feeders, assessment of venous drainage, and recognition of associated injuries, such as pseudoaneurysms or vessel irregularities.

A stepwise multimodality imaging approach improves diagnostic confidence, enables precise treatment planning, and leads to better outcomes with timely endovascular intervention, underscoring the need for a low threshold for imaging and a high index of suspicion, particularly following cervical trauma or vascular instrumentation.

## References

[1] Heuer GG, Gabel BC, Bhowmick DA, Stiefel MF, Hurst RW, Schuster JM (2008). Symptomatic high-flow arteriovenous fistula after a C-2 fracture. Case report. J Neurosurg Spine.

[2] Stock U, Link J, Dütschke P (1996). Iatrogenic vertebrojugular arteriovenous fistula. Anaesthesia.

[3] Kypson AP, Wentzensen N, Georgiade GS, Vaslef SN (2000). Traumatic vertebrojugular arteriovenous fistula: case report. J Trauma.

[4] Shirakawa M, Nishioka T, Yamashita K, Maeda Y, Arita N (2008). Traumatic vertebro-vertebral arteriovenous fistula manifesting as radiculopathy. Case report. Neurol Med Chir (Tokyo).

[5] Madoz A, Desal H, Auffray-Calvier E, Isnard J, Liberge R, Taverneau C, De Kersaint-Gilly A (2006). Vertebrovertebral arteriovenous fistula diagnosis and treatment: report of 8 cases and review of the literature (Article in French). J Neuroradiol.

[6] López-Medina A, López-Vidaur I, Villoria R, Fernández-Cantón G, Martín-Gómez JI (1995). Arteriovenous fistulas in the neck: diagnosis with color Doppler sonography. J Ultrasound Med.

[7] Mi HX, Guo CM, Chen SL, Zhang J, Gao Z, Hongxin L, Han J (2025). Case report: endovascular therapy for an iatrogenic vertebrojugular arteriovenous fistula and pseudoaneurysm induced by multiple vascular procedures. Front Cardiovasc Med.

[8] Melge PP, Kothurkar AA, Date SV (2025). Traumatic Vertebrojugular arteriovenous fistula: a rare presentation of penetrating traumatic injury - a case report and review of literature. Indian J Vasc Endovasc Surg.

[9] Yoshihara T, Yamamura N, Okubata H (2025). High-energy blunt traumatic vertebro-vertebral arteriovenous fistula with retropharyngeal hematoma: a case report. J Med Case Rep.

